# Supplementary material for: Hydration Free Energies of Linear Alkanes: Systematic Deviations in Common Water Models and Their Correction
Source: J Phys Chem B. 2026 Apr 16;130(17):4636–44. doi: 10.1021/acs.jpcb.5c07832 (PMC13137253; doi:10.1021/acs.jpcb.5c07832)
Supplement: Supplementary file 1 [file jp5c07832_si_001.pdf]

# Supporting Information: Hydration Free Energies of Linear Alkanes: Systematic Deviations in Common Water Models and Their Correction

Yalda Ramezani and Sumit Sharma[1*]

*Department of Chemical and Biomolecular Engineering, Ohio University, Athens, OH - 45701*

E-mail: sharmas@ohio.edu

Table S1: Details of the Alkane-Water Simulation System. Three system sizes are used depending on the alkane length. The simulation system volume fluctuates to maintain a constant pressure.

| Alkane | box length [Å] | Number of Water Molecules |
|---|---|---|
| $C_1$-$C_8$ | 36 | 1500 |
| $C_9$-$C_{14}$ | 46 | 3390 |
| $C_{15}$-$C_{20}$ | 56 | 6440 |

Table S2 compares the $\Delta G_{hyd}$ estimated in this work at 300 K using the TraPPE force field and the TIP4P/2005 and SPC/E water models with the previous estimates by Chen (2000),[1] Xue (2018),[2] and Ashbaugh (2011).[3] Our estimates for TraPPE + TIP4P/2005 match well with those of Ashbaugh (2011) and Xue (2018) with the maximum deviation of 1.65 kJ/mol observed for $C_9$ with Xue (2018). The results from the OPLS + TIP4P are also within 0.7 kJ/mol of our estimates for TraPPE + TIP4P/2005. Our results using the SPC/E water model show the largest deviation of 1.5 kJ/mol for $C_8$ when compared to Xue (2018). Overall, this shows that our estimates are in good agreement with the previous studies.

[1]Corresponding author: Sumit Sharma

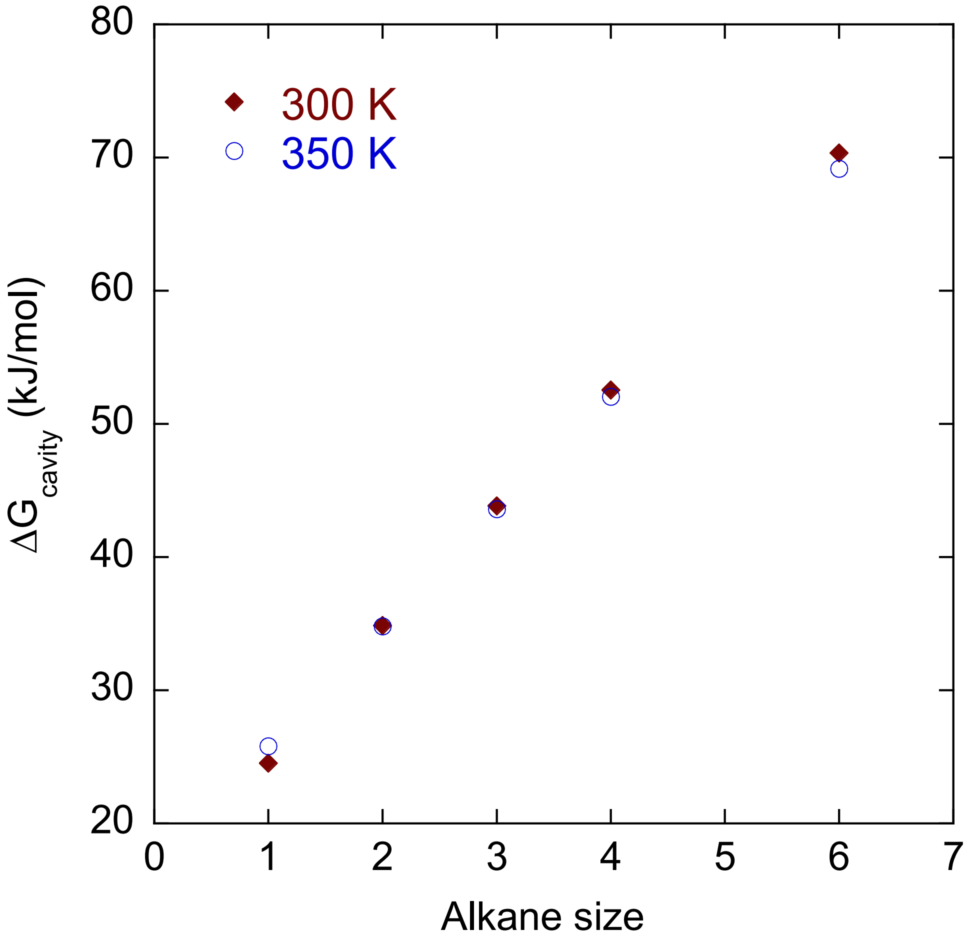


Figure S1: Comparison of cavity free energy $\Delta G_{cavity}$ at 300 K and 350 K for different alkanes. The $\Delta G_{cavity}$ does not change over this temperature range.

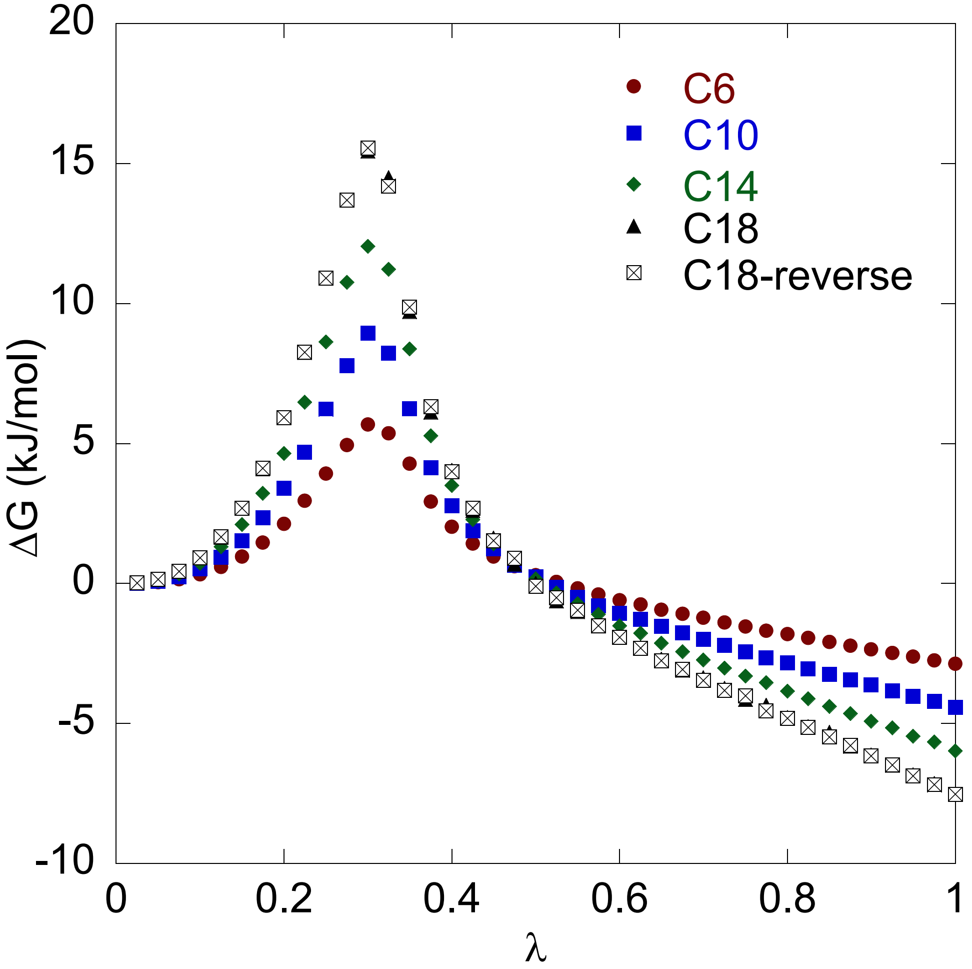


Figure S2: Free energy change, $\Delta G$ of selected alkanes as a function of the FEP parameter, $\lambda$ at 300 K. For C18, the $\Delta G$ of the reverse path is also plotted to show the overlap between the forward and the reverse FEP simulation results.

Table S2: Comparison of hydration free energy obtained in this work at 300 K with previous studies. The estimated error in our calculation, determined from three independent simulations, is 0.05 kJ/mol up to $C_6$, 0.1 kJ/mol up to $C_{11}$, and 0.4 kJ/mol for longer alkanes.

| Alkane | This work | | Chen (2000)[1] | Xue (2018)[2] | | Ashbaugh (2011)[3] |
|---|---|---|---|---|---|---|
| | TraPPE+TIP4P/2005 | TraPPE+SPC/E | OPLS+TIP4P | TraPPE+TIP4P/2005 | TraPPE+SPC/E | TraPPE+TIP4P/2005 |
| 1 | 9.26 | 9.39 | 9.40 | | | 9.33 |
| 2 | 8.82 | 9.28 | 8.20 | | | 8.66 |
| 3 | 9.75 | 9.94 | 9.70 | | | 9.67 |
| 4 | 10.61 | 11.16 | 10.70 | | | 10.33 |
| 5 | 11.69 | 12.19 | | 11.79 | 11.05 | |
| 6 | 12.62 | 13.11 | | 12.99 | 12.12 | |
| 7 | 13.68 | 14.58 | | 14.38 | 13.13 | |
| 8 | 14.64 | 15.69 | | 15.91 | 14.19 | |
| 9 | 16.10 | 16.62 | | 17.75 | 15.30 | |

Table S3: Hydration free energies (kJ/mol) of linear alkanes from methane ($C_1$) to eicosane ($C_{20}$) at 300 K using different water models with alkane-water interaction parameters calculated using Lorentz-Berthelot mixing rules. The estimated error in our calculation, determined from three independent simulations, is 0.05 kJ/mol up to $C_6$, 0.1 kJ/mol up to $C_{11}$, and 0.4 kJ/mol for longer alkanes.

| Alkane | Experiments | Group Contribution | TIP4P/2005 | OPC | SPC/E | OPC3 | HH-alkane | Shifted HH-alkane |
|---|---|---|---|---|---|---|---|---|
| 1 | 8.37 | | 9.26 | 9.72 | 9.39 | 9.23 | 8.63 | 8.48 |
| 2 | 7.66 | | 8.82 | 8.73 | 9.28 | 8.81 | 7.94 | 7.99 |
| 3 | 8.18 | | 9.75 | 9.67 | 9.94 | 9.90 | 8.60 | 8.88 |
| 4 | 8.70 | | 10.61 | 10.59 | 11.16 | 11.19 | 9.02 | 9.54 |
| 5 | 9.76 | | 11.69 | 11.67 | 12.19 | 12.13 | 10.01 | 10.09 |
| 6 | 10.40 | | 12.62 | 12.33 | 13.10 | 13.39 | 10.83 | 11.12 |
| 7 | 10.96 | | 13.68 | 13.59 | 14.58 | 14.64 | 11.26 | 11.89 |
| 8 | 12.10 | | 14.64 | 14.42 | 15.69 | 15.68 | 11.84 | 13.00 |
| 9 | | 12.58 | 16.10 | 15.17 | 16.62 | 16.89 | 12.83 | 13.93 |
| 10 | | 13.32 | 16.92 | 16.09 | 18.08 | 18.19 | 13.60 | 14.76 |
| 11 | | 14.06 | 17.94 | 17.07 | 19.47 | 19.35 | 14.46 | 15.70 |
| 12 | | 14.80 | 18.70 | 17.92 | 20.15 | 20.58 | 15.39 | 17.11 |
| 13 | | 15.54 | 19.85 | 19.24 | 21.63 | 21.61 | 16.24 | 17.11 |
| 14 | | 16.28 | 20.44 | 20.16 | 22.95 | 23.07 | 16.67 | 18.27 |
| 15 | | 17.02 | 21.69 | 21.73 | 24.35 | 24.13 | 17.40 | 19.43 |
| 16 | | 17.76 | 23.03 | 22.11 | 25.15 | 25.68 | 18.17 | 20.75 |
| 17 | | 18.50 | 24.57 | 24.00 | 26.58 | 27.08 | 18.85 | 21.19 |
| 18 | | 19.24 | 25.03 | 24.61 | 27.64 | 27.72 | 20.03 | 22.04 |
| 19 | | 19.98 | 26.97 | 25.91 | 29.25 | 28.74 | 21.55 | 23.10 |
| 20 | | 20.72 | 28.03 | 27.20 | 30.49 | 30.97 | 21.69 | 23.80 |

Table S4: Hydration free energies (kJ/mol) of alkanes at 300 K with different water models with updated alkane-water well-depth parameter ($\epsilon$).

| Alkane | TIP4P/2005 | OPC | SPC/E | OPC3 |
|---|---|---|---|---|
| 1 | 8.84 | 9.11 | 8.53 | 8.85 |
| 2 | 7.87 | 7.95 | 7.58 | 7.68 |
| 6 | 10.35 | 10.10 | 10.34 | 10.43 |
| 10 | 13.00 | 13.12 | 13.47 | 13.69 |
| 14 | 16.20 | 15.73 | 17.34 | 17.36 |
| 18 | 19.53 | 19.20 | 19.89 | 21.10 |
| 20 | 22.13 | 20.62 | 22.09 | 22.03 |

Table S5: Hydration free energy (in $kT$) of alkanes at temperatures ranging from 290 to 350 K calculated for different water models with updated alkane-water well-depth parameter ($\epsilon$). The estimated error is of the order of 0.05 $kT$.

| Model | T (K) | C1 | C2 | C3 | C4 |
|---|---|---|---|---|---|
| | 270 | 2.94 | 2.12 | 2.25 | 2.27 |
| | 290 | 3.33 | 2.84 | 2.98 | 3.24 |
| | 300 | 3.54 | 3.16 | 3.32 | 3.56 |
| Tip4p2005 | 310 | 3.59 | 3.27 | 3.63 | 3.82 |
| | 330 | 3.74 | 3.53 | 3.92 | 4.25 |
| | 350 | 3.80 | 3.65 | 4.11 | 4.50 |
| | 370 | 3.75 | 3.70 | 4.22 | 4.55 |
| | 270 | 3.05 | 2.46 | 2.53 | 2.56 |
| | 290 | 3.33 | 2.91 | 3.15 | 3.39 |
| | 300 | 3.42 | 3.08 | 3.40 | 3.57 |
| SPC/E | 310 | 3.51 | 3.22 | 3.52 | 3.83 |
| | 330 | 3.56 | 3.43 | 3.74 | 4.06 |
| | 350 | 3.63 | 3.51 | 3.84 | 4.25 |
| | 370 | 3.56 | 3.44 | 3.93 | 4.26 |
| | 270 | 3.08 | 2.17 | 2.19 | 2.28 |
| | 290 | 3.52 | 2.93 | 3.03 | 3.27 |
| | 300 | 3.65 | 3.19 | 3.43 | 3.62 |
| OPC | 310 | 3.76 | 3.41 | 3.71 | 3.90 |
| | 330 | 3.98 | 3.68 | 4.09 | 4.41 |
| | 350 | 4.01 | 3.91 | 4.26 | 4.65 |
| | 370 | 3.95 | 3.93 | 4.39 | 4.91 |
| | 270 | 2.95 | 2.37 | 2.51 | 2.53 |
| | 290 | 3.34 | 2.84 | 3.13 | 3.35 |
| | 300 | 3.48 | 3.08 | 3.27 | 3.54 |
| OPC3 | 310 | 3.54 | 3.23 | 3.53 | 3.78 |
| | 330 | 3.66 | 3.49 | 3.84 | 4.17 |
| | 350 | 3.69 | 3.64 | 4.02 | 4.38 |
| | 370 | 3.63 | 3.60 | 4.01 | 4.40 |
| | 270 | | | | |
| | 290 | 3.24 | 2.88 | 3.06 | 3.23 |
| | 300 | 3.41 | 3.13 | 3.35 | 3.57 |
| Expt. | 310 | 3.53 | 3.33 | 3.58 | 3.84 |
| | 330 | 3.69 | 3.58 | 3.86 | 4.20 |
| | 350 | 3.74 | 3.67 | 3.95 | 4.36 |
| | 370 | | | | |

Table S6: Hydration free energies (kJ/mol) of alkanes computed using the General Amber Force Field and TIP4P/2005 and SPC/E water models at 300 K.

| Alkane | TIP4P/2005 | SPC/E |
|---|---|---|
| 1 | 9.70 | 9.80 |
| 2 | 9.50 | 10.01 |
| 5 | 10.28 | 11.21 |
| 8 | 11.85 | 13.11 |
| 11 | 12.83 | 15.67 |
| 14 | 14.89 | 17.88 |
| 17 | 16.96 | 19.99 |

# References

(1) Chen, B.; Siepmann, J. I. A novel Monte Carlo algorithm for simulating strongly associating fluids: Applications to water, hydrogen fluoride, and acetic acid. *The Journal of Physical Chemistry B* **2000**, *104*, 8725–8734.

(2) Xue, B.; Harwood, D. B.; Chen, J. L.; Siepmann, J. I. Monte Carlo Simulations of Fluid Phase Equilibria and Interfacial Properties for Water/Alkane Mixtures: An Assessment of Nonpolarizable Water Models and of Departures from the Lorentz–Berthelot Combining Rules. *The Journal of Physical Chemistry B* **2018**, *122*, 7617–7627.

(3) Ashbaugh, H. S.; Liu, L.; Surampudi, L. N. Optimization of linear and branched alkane interactions with water to simulate hydrophobic hydration. *The Journal of chemical physics* **2011**, *135*.
